# Nurses Boxing with Patients

**Published:** 1917-04-07

**Authors:** H. E. Bruce Porter

**Affiliations:** Lt. Col. Commanding 3rd London General Hospital, S.W.


### Nurses Boxing with Patients.

"An Evil of Illustrated Papers."

*To the Editor of* The Hospital.

Sir,—I regret to notice in the Sunday pictorial papers two lady clerks and a V.A.D. of this hospital photographed as if boxing with patients. This episode is a gross breach of a privilege given to a photographer to take a Police Assault at Arms; after leaving the hall where this was taking place he met these stupid girls and persuaded them to pose. We all regret the bad taste of these ladies and their folly in allowing themselves to be photographed, and they have been dealt with. I am writing to you in case you should have letters sent you from outside criticising these photographs, as, indeed, they would have every right to do, and in the hope that you will forward this explanation to whoever writes to you. Thanking you in anticipation of your courtesy.—Yours very truly,

H. E. Bruce Porter, Lt. Col.
Commanding 3rd London General Hospital, S.W.

April 2, 1917.

[It is regrettable to note the repeated evils which too often arise from the defective editing of cheap illustrated papers.—Ed. The Hospital.]

